# Metabolic engineering of *Thermoanaerobacterium* AK17 for increased ethanol production in seaweed hydrolysate

**DOI:** 10.1186/s13068-023-02388-y

**Published:** 2023-09-11

**Authors:** Antoine Moenaert, Bryndís Bjornsdottir, Einar Baldvin Haraldsson, Leila Allahgholi, Anna Zieri, Isabella Zangl, Sigríður Sigurðardóttir, Jóhann Örlygsson, Eva Nordberg Karlsson, Ólafur H. Friðjónsson, Guðmundur Óli Hreggviðsson

**Affiliations:** 1 Department of Biotechnology, Matís Ohf, Reykjavík, Iceland; 2 Faculty of Life and Environmental Sciences, University of Iceland, Reykjavík, Iceland; 3 Biotechnology, Department of Chemistry, Lund University, Lund, Sweden; 4 IMC University of Applied Sciences Krems, Krems, Austria; 5 Faculty of Natural Resource Sciences, University of Akureyri, Akureyri, Iceland

**Keywords:** Macroalgae, Seaweed, Biorefinery, Fermentation, Biofuel, Ethanol, Metabolic engineering, Thermophile

## Abstract

Sustainably produced renewable biomass has the potential to replace fossil-based feedstocks, for generation of biobased fuels and chemicals of industrial interest, in biorefineries. In this context, seaweeds contain a large fraction of carbohydrates that are a promising source for enzymatic and/or microbial biorefinery conversions. The thermoanaerobe *Thermoanaerobacterium* AK17 is a versatile fermentative bacterium producing ethanol, acetate and lactate from various sugars. In this study, strain AK17 was engineered for more efficient production of ethanol by knocking out the lactate and acetate side-product pathways. This was successfully achieved, but the strain reverted to acetate production by recruiting enzymes from the butyrate pathway. Subsequently this pathway was knocked out and the resultant strain AK17_M6 could produce ethanol close to the maximum theoretical yield (90%), leading to a 1.5-fold increase in production compared to the wild-type strain. Strain AK17 was also shown to successfully ferment brown seaweed hydrolysate from *Laminaria digitata* to ethanol in a comparatively high yield of 0.45 g/g substrate, with the primary carbon sources for the fermentations being mannitol, laminarin-derived glucose and short laminari-oligosaccharides. As strain AK17 was successfully engineered and has a wide carbohydrate utilization range that includes mannitol from brown seaweed, as well as hexoses and pentoses found in both seaweeds and lignocellulose, the new strain AK17_M6 obtained in this study is an interesting candidate for production of ethanol from both second and third generations biomass.

**Supplementary Information:**

The online version contains supplementary material available at 10.1186/s13068-023-02388-y.

## Background

Utilization of renewable biomass as feedstocks for production of energy carriers (biofuels) is of major interest, the most common product being ethanol, which is used both as a biofuel and as a top platform chemical [1]. Ethanol is produced from sugar-rich crops [2, 3]. In Europe, it is mainly produced from grains and sugar beet derivatives [4], whereas from sugarcane in Brazil [5] and from corn in the United States [6]. These types of biomasses are efficiently converted to ethanol by conventional fermenting organisms, like yeast (e.g., *Saccharomyces cerevisiae*) [7]. The production, however, depends on the use of first generation biomass, rich in starch that requires the use of arable land in competition with its utilization for generation of food and feed. It also competes for water utilization and requires use of fertilizers and pesticides, which reduces its positive environmental impact [8]. Therefore, studies have focused on the utilization of more sustainable biomass such as lignocellulose from forest and agricultural side streams, industrial and consumer wastes, as well as micro- and macroalgae that do not compete for arable land.

Macroalgae are underutilized feedstocks for industrial conversions. They contain polysaccharides, of vastly different structures than those typically found in terrestrial plant biomass. Terrestrial plant polysaccharides can, after hydrolysis, be converted to various biobased products by conventional fermentative microorganisms such as yeast. On the other hand, macro-algal polysaccharides require a different set-up of enzymes and pathways for their conversion [9]. Biorefinery utilization of macroalgae therefore needs new fermentative organisms and/or modified conventional organisms that can utilize macro-algal carbohydrates. The efficiency and yields of new fermentative microorganisms may, however, still be low for industrialization. To improve microbial ethanol production on complex biomasses, studies have focused on two strategies: either implementing metabolic pathways in *E. coli* or yeast to allow them to grow on complex substrates [10, 11], or using genetic engineering to improve ethanol production in strains that have a wide substrate range, such as certain thermophilic anaerobic bacteria [12–14].

Species of the thermophilic genus *Thermoanaerobacterium* and related genera have been studied intensively in recent years, because of their natural and relatively large substrate range and high fermentative ethanol yields. They have natural genetic competence and therefore can be relatively easily engineered for improved metabolic efficiency. For example, *Thermoanaerobacterium thermosaccharolyticum* has been engineered to produce ethanol at 92% theoretical yield [12, 15] compared to *Clostridium thermocellum* at 75% yield [13, 16]. Genetic tools have been developed during the last 10 years, to engineer *Thermoanaerobacterium* species and other related thermoanaerobes [17–19]. Replicative plasmids have been developed for efficient transformation and rapid expression of heterologous genes in *Thermoanaerobacterium* and *Thermoanaerobacter* species [20].

The main threshold for genetic engineering of thermophiles has been the thermolability of many antibiotics as well as marker proteins [21]. This leaves few alternatives for use in thermophiles, which drastically reduces the scope of genetic manipulations of these organisms. So far, erythromycin and kanamycin can be used as selection as they can be produced in active form in thermophiles up to 65 °C. In addition, both positive and negative selection markers have been used to enable recycling of the same antibiotic marker and to recreate marker-free strains. A well-known system of this type involves the utilization of the gene encoding the enzyme orotidine 5-phosphate decarboxylase (PyrF) [18, 22, 23]. This enzyme is involved in the de novo biosynthesis of pyrimidine, but is also a target for the antimetabolite 5-fluoroorotic acid (5-FOA) [22]. Cells lacking *pyrF* are unable to produce uracil and are resistant to 5-FOA. This creates an opportunity for the selection or counterselection of cells with ectopic expression of *pyrF*. For instance, this system has been used in *Clostridium thermocellum* to delete the phospho-trans-acetylase gene (*pta*) [24].

The investigation reported here focuses on *Thermoanaerobacterium* AK17, isolated from a hot spring in the Krafla volcano in northern Iceland. It is closely related to *Thermoanaerobacterium xylanolyticum* LX-11 (99% identity based on 16SrRNA) [25]. Strain AK17 is a fermentative thermoanaerobe, growing optimally at 60 °C and pH 6.0 [26]. Early experiments suggested that this strain could ferment a wide spectrum of carbohydrates and it maximally produced 0.25 and 0.39 g ethanol per g of grass or cellulose, respectively [27]. It could also use xylose and arabinose, the dominant pentoses in hydrolysates of lignocellulosic biomass, as carbon sources for growth [25, 28]. End-products were ethanol, acetate, lactate, and hydrogen, with a maximum ethanol yield of 1.5 mol/mol of glucose (75% of the maximum theoretical yield). Recently, the strain was also evaluated for its potential to ferment seaweed hydrolysate to produce bioethanol [29].

In this study, the aim was to knock out both lactate and acetate production pathways to make ethanol the only fermentation product. This was successfully achieved, but the new engineered strain was not stable and reverted to producing acetate again. After identifying the enzymes responsible for acetate formation, a new strain was engineered by knocking out genes in the responsible pathway. Subsequently, this strain produced ethanol as the only fermentation product. Finally, it was shown that the strain could grow on relatively crude brown seaweed hydrolysates from *Laminaria digitata,* fermenting the main digestible carbon sources (mannitol and laminarin) in the hydrolysate to ethanol.

## Material and methods

### Fermentation conditions and media

In the studies performed, strain *Thermoanaerobacterium* AK17 was cultivated on BM medium [28] containing (in g/L): 5.5 NaH_2_PO_4_, 0.6 Na_2_HPO_4_, 0.6 KH_2_PO_4_, 0.3 NH_4_Cl, 0.3 NaCl, 0.1 CaCl_2_·2H_2_O, 0.1 MgCl_2_·6H_2_O, 0.1 resazurin, 2.0 yeast extract (YE, Difco). The pH was adjusted to 5.6, so that after addition of the reducing agents, the final pH was around 6.0. A micronutrient-solution was prepared as follows (in g/L): 2.0 FeCl_2_·4H_2_O, 0.05 H_3_BO_3_, 0.05 ZnCl_2_, 0.038 CuCl_2_·2H_2_O, 0.041 MnCl_2_·2H_2_O, 0.05 (NH_4_)_6_Mo_7_O_24_·4H_2_O, 0.05 AlCl_3_, 0.05 CoCl_2_·6H_2_O, 0.05 NiCl_2_ · 6H_2_O, 0.5 EDTA, 0.026 Na_2_SeO_3_·5H_2_O, 0.033 NaWO_4_·2H_2_O.A vitamin-solution was also prepared following the DSMZ medium No141 (German Collection of Microorganisms and Cell Cultures). Tubes and flasks containing media were closed with rubber stoppers and caps, then flushed with N_2_ for 5 min to ensure the removal of oxygen, and autoclaved (121 °C, 20 min). Finally, micronutrients (1.0 mL/L medium), vitamins (1.0 mL/L medium), carbon source (glucose, 4.5 g/L) and reducing agent (3.0 mM cysteine hydrochloride + 3.0 mM Na_2_S) were added prior to inoculation. The medium was inoculated with 2% (v/v) culture and incubated overnight (or until further mentioned) at 60 °C in the dark without shaking.

### Selective conditions and media

For antibiotic selection, strain AK17 was grown on BM medium supplemented with either 50 µg/mL kanamycin sulfate (Sigma) or 1.0 µg/mL erythromycin (Sigma). For the selection of resistance to 5-fluoroorotic acid (5-FOA), the strain was grown on BM medium supplemented with 1.0 mg/mL of 5-FOA (ThermoFisher) and 600 µL/L 24.5% HCl to adjust the final pH to 5.0 (working pH for 5-FOA and sub-optimal growth for AK17). For the selection of uracil autotrophy, the strain was cultivated on a modified DSMZ 122 medium [30], which contained (in g/L): 1.46 KH_2_PO_4_, 1.80 K_2_HPO_4_, 6.0 Na-β-glycerophosphate, 1.3 (NH_4_)_2_SO_4_, 0.13 CaCl_2_·2H_2_O, 2.6 MgCl_2_∙6H_2_O, 0.8 NaHCO_3_, 0.1 resazurin and the yeast extract was replaced with 2X RPMI 1640 vitamins (Sigma R7256) and 1X minimal essential medium (MEM) amino acids (Sigma M5550). The pH was adjusted to 5.6, and glucose, vitamins, micronutrients and reducing agents were added as described above, to reach a final pH of 6.0.

### Fermentation on hydrolysates

Growth on a brown seaweed hydrolysate was also investigated. The hydrolysate was obtained from wild grown sugar-rich *Laminaria digitata* collected in August 2014 off the coast of Denmark, as previously described by Hou and co-workers [31]. Briefly, the dried seaweed was processed by partial enzymatic hydrolysis at the Danish Technological Institute pilot scale (600L) facility, resulting in conversion of the glucans to both oligosaccharides and monosaccharides. The carbohydrate content of the *L. digitata* hydrolysate was (in g/L): 10.0 glucose, 5.0 mannitol, 10.0 oligoglucans, and 1.2 lactic acid, where lactic acid was judged as a contamination introduced during the enzymatic processing, as described by Hou and co-workers [31]. Strain AK17 was first cultured on BM medium, then two percent of exponentially grown culture was transferred to fresh BM medium containing 50 or 100% (v/v) seaweed hydrolysate (pH adjusted to 6.5 with 1 M NaOH, supplemented with 0.2% YE). Vitamins and reducing agents were added prior to inoculation, as described for BM medium above. Cultivations were carried out in anaerobic conditions, using closed flasks (118 mL) with 50 mL working volume, at 60 °C for 4 days. Samples were taken at different time intervals to monitor the ethanol production, and cell density was measured at the start (0 h) and end (24 h) of the cultivation.

### Strains and construction of plasmids

*Escherichia coli* DH5 (NEB) was used as the host for recombinant DNA manipulation, and bacteria were grown in Lysogeny Broth (LB) (BD Difco), with antibiotic (ampicillin at 100 µg/mL or kanamycin at 30 µg/mL).

All plasmids and strains used or constructed during this study are listed in Table 1. Polymerase chain reaction (PCR) was performed using the high-fidelity Q5 Taq polymerase (NEB). The genome of strain AK17 was sequenced using MiSeq and the genes encoding lactate dehydrogenase (*ldh*), acetate kinase (*ack*) and phosphate acetyltransferase (*pta*) were identified by amino acid identity comparison with previously described homologues using BLAST. The *ack* and *pta* genes were adjacent to one another in the genome. Two deletion cassettes were constructed to eliminate the *ldh* and *ack/pta* genes, using primers listed in Table S1 and standard PCR and cloning techniques. The cassettes contained the 5´ and 3´ regions (300 to 500 pb) up- and down-stream of the *ldh* and *ack/pta* genes with resistance genes between them, amplified and spliced together using overhang extensions. The pUC_18 was used as the backbone, and cut bluntly with SmaI DNAse, before ligation with the deletion cassettes.

**Table 1:** List of strains and plasmids used and developed in this study

Plasmid/strain ID	Description	Source
pUC_18	Cloning vector	Thermo Fisher
pUC_1801	Vector for insertion of *erm* into *ldh* locus	This study
pUC_1802	Vector for insertion of *kan* into *ack/pta* loci	This study
pUC_1803	Vector for removal of *pyrF*	This study
pUC_1804	Vector for insertion of *pyrF* into *ldh* locus	This study
pUC_1805	Vector for the removal of *pyrF* from *ldh* locus	This study
pUC_1806	Vector for insertion of *pyrF* into *bk/ptb* loci	This study
NEB10	*E.coli* cloning strain	New England Biolab
AK17_WT	*Thermoanaerobacterium* AK17 Wild type	Matís strain collection
AK17_M1	AK17_WT1 + pUC1801: Δldh:erm	This study
AK17_M2	AK17_WT1 + pUC1802: Δack/pta:kan	This study
AK17_M3	AK17_M1 + pUC1802: Δack/pta:kan Δldh:erm	This study
AK17_M3ad	AK17_M3 after several (> 10) passages	This study
AK17_M4	AK17_M3 + pUC1803: Δack/pta:kan Δldh:erm ΔpyrF	This study
AK17_M5	AK17_M4 + pUC1804: Δack/pta:kan Δldh:pyrF	This study
AK17_M6	AK17_M5 + pUC1805: Δack/pta:kan Δldh ΔpyrF	This study
AK17_M7	AK17_M6 + pUC1806: Δack/pta:kan Δldh Δbk/ptb:pyrF	This study

The *ldh* gene was replaced with a thermostable erythromycin resistance gene (*erm*), originating from *Enterococcus faecalis* (GenBank: AB247327.1). The gene was codon optimized for use in strain AK17 and synthesized (Eurofins MWG Operon) before being cloned to obtain the plasmid pUC_1801. The adjacent *ack/pta* genes were replaced with a thermostable kanamycin resistance gene (*kan*) originating from *Bacillus sp*. (GenBank: NG_047372.1). The gene was then amplified before being cloned to obtain the plasmid pUC_1802. Construction verification was performed by PCR and sequencing, using universal M13 primers. All primers used for construction of the plasmids are listed in Additional file 1: Table S1.

In order to perform other genetic manipulations, the pyrF system was investigated to counter the lack of thermostable antibiotic resistance genes. The pyrF system was developed using the *pyrF* gene, responsible for uracil synthesis [24]. In short, *pyrF* was first knocked out to generate an uracil auxotrophic strain. Transformants were selected using 5-fluoroorotic acid (5-FOA) in a medium with uracil. 5-FOA is a toxic uracil analogue for PyrF, allowing only strains without *pyrF* to survive. A new cassette was then constructed to insert *pyrF* in a target gene for knockout. Reinserting the *pyrF* gene regenerated the uracil prototrophy (Additional file 2: Figure S1). Hence, transformants could be selected using medium without uracil. New strains could be further manipulated by redeleting/reinserting the *pyrF* gene.

In the present study, a plasmid was constructed containing the flanking region of *pyrF* in the genome of strain AK17 (pUC_1803) to remove *pyrF*. Then another plasmid was designed containing the *pyrF* gene between the flanking regions of the *ldh* gene (pUC_1804), to remove the *erm* gene used primarily to knock out the *ldh* gene (see above). Subsequently, a plasmid was designed only containing the flanking regions of the *ldh* genes (pUC_1805), to delete the pyrF gene. Lastly, a knockout plasmid was designed containing the *pyrF* gene between the flanking regions of the *bk/ptb* genes (pUC_1806).

### Transformation of AK17

Cells of strain AK17 were grown on BM medium at 60 °C until reaching an OD of ~ 0.8 at 600 nm. The cells from 15 mL of culture were then harvested by centrifugation in anaerobic Hungate tubes (2500 × g, 20 min, 4 °C) and washed three times with 8 mL of 10% glycerol (flushed with N_2_) under the same conditions. The cells were suspended in 0.1 mL of dH_2_O and 40 µL transferred to a prechilled 0.1 cm electroporation cuvette containing approximately 1 µg plasmid DNA. The transformation was carried out using single pulse electroporation (GenePulser Xcell, BioRad, 1.25 kV, 400 Ω, 25 mF) with a time constant between 8 and 10 ms. Control cells were electroporated without the plasmid. Cells were then immediately transferred to Hungate tubes with 5 mL of BM medium and allowed to recover at 55 °C for up to 16 h. Cells were then plated onto selective BM agarose medium containing the appropriate selection marker (50 µg/mL kanamycin, 1.0 µg/mL erythromycin or 1.0 mg/mL 5-FOA), or onto BM for control. The plates were incubated anaerobically at 55 °C for 2/3 days.

### Protein extraction and enzymatic assay

The different engineered strains obtained in this study, AK17_M3 and AK17_M3ad along with the wild-type strain AK17_WT, were cultivated in 300-mL anaerobic flasks with 150 mL working volume until reaching maximum OD_600_, and cells were harvested by centrifugation at 2500 × g for 15 min. Cell biomass was estimated by weighing the cell pellet after centrifugation, then resuspended in 50 mM, pH 6.2 KPO4 buffer to achieve a cell biomass suspension of 0.1 g/mL. Cell suspensions were sonicated 2 × 3 min (DUTY cycles) using a sonicator (Branson Sonifier 250), then centrifuged at 2500 × g for 20 min to separate the cell debris from the soluble fraction containing the enzymes (also called crude extract). Total protein concentrations of the crude extracts were determined using the Quick Start™ Bradford Protein Assay (Bio-Rad), with bovine serum albumin as standard. Crude extracts were aliquoted in Eppendorf tubes, flash frozen in liquid nitrogen and stored at − 80 °C until further use.

The activity of the phosphotransbutyrylase (PTB) and phosphotransacetylase (PTA) were assessed by the release of CoA from butyryl-CoA and acetyl-CoA in consequent formation of acetyl phosphate and butanoyl phosphate, following the method developed by Andersch and co-workers, and Cary and co-workers [32, 33]. Briefly, free CoA was reacted with DTNB (Ellman's reagent), and the absorbance was read at 412 nm. A standard curve was created using L-cysteine, 0.0–6.0 mM (0.0–1.8 μmol). The assay was carried out at 55 °C for 20 min on a 96-well flat bottom plate (Nunc), in 300 mM KPO_4_ buffer, pH 7.29 (pH 7.20 at 55 °C). Specific activity was defined as micromole of CoA released per minute relative to milligramme of total protein (U/mg). DTNB solution was prepared in 100 mM KPO_4_, pH 7.2, 0.1 mM EDTA.

The activity of the acetate kinase (AK) and the butyrate kinase (BK) was assessed by adapting the method developed by Fowler and co-workers for both enzymes [34]. Briefly, the assay is based on direct determination of the consumption of acetyl/butanoyl phosphate. After the enzymatic reaction, the remaining acetyl/butanoyl phosphate is converted to a ferric hydroxamate complex which is absorbed at 540 nm. Two standard curves were made, with acetyl phosphate and butanoyl phosphate, 0.00–0.90 μmol (0.0–3.0 mM). The assay was performed at 55 °C for 20 min in a 96-well flat bottom plate (Nunc), in 100 mM Tris buffer pH 8.39 (7.4 at 55 °C). After addition of hydroxylamine at 60 °C for 5 min, the reaction was stopped by addition of the development solution (1% FeCl_3_, 1 M HCl and 2% trichloroacetic acid). The absorbance was then read at 540 nm. The specific activity of the reaction was defined as micromole of consumed acetyl- and butanoyl phosphate per minute relative to milligramme of total protein (U/mg).

### Glucose, mannitol, organic acids, and ethanol measurements

Consumption of glucose, mannitol, and production of lactic acid, acetic acid, and ethanol by strain AK17 and the derived mutant strains were analysed by high performance liquid chromatography (HPLC). The samples for HPLC analysis were filtered through a 0.2 µm filter (Phenomenex) prior injection. Glucose, mannitol, acetic acid, lactic acid, and ethanol were quantified using a Dionex 2000 HPLC system (Dionex, Idstein, Germany) with a Rezex ROA-Organic Acid H + (8%, Phenomenex, Aschaffenburg, Germany) and a RI-101 detector (Shodex, München, Germany). Separation was performed at a column temperature of 60 °C with 0,2 mM sulfuric acid (Carl Roth, Karlsruhe, Germany) as eluent at a flow rate of 600 μl/min for 30 min. Quantification was carried out using external standards with HPLC grade (Merck, Darmstadt, Germany; Sigma-Aldrich, St. Louis, USA) and the Chromeleon Evaluation Software version 6.80 (Dionex, Idstein, Germany).

### Total monosaccharides analysis by HPAEC-PAD

Polysaccharides were hydrolysed to monosaccharides by a two-step acid hydrolysis process [35]. Twenty-five milligrammes of the samples were mixed with 250 µL of sulfuric acid 72% (w/w) (Thermo Scientific™) in Pyrex tubes with screw cap. The tubes were incubated at 30 °C and 150 rpm shaking for 1 h. Afterward, 7 mL of milliQ-water was added to the tubes to reach the acid concentration of 4% (w/w). The samples were vortexed and autoclaved for 1 h. After cooling down, the samples were centrifuged at 3000 × g (Sigma 3-16PK centrifuge, Germany) for 10 min to remove the solid particles. The hydrolysate was then neutralized by barium hydroxide (0.1 M).

To analyse the monosaccharides obtained after this hydrolysis, samples were diluted with milliQ-water and filtered through a 0.2 µm syringe filter. Monosaccharides were analysed by a High Performance Anion Exchange Chromatography equipped with Pulsed Amperometric Detection (HPAEC-PAD) system (Thermo Fisher Scientific, USA) using a Carbopac PA-20 column coupled with a guard column (Thermo Fisher Scientific, USA). Neutral sugars were separated under isocratic condition using Milli-Q-water (A), 2 mM sodium hydroxide (B) and 200 mM sodium hydroxide (C) as eluents. The elution was performed using an eluent mixture of 62.5% (A) and 37.5% (B) for 25 min. Separation was carried out at a flow rate of 0.5 mL/min, and the column and compartment temperature was kept at 30 °C [36]. The concentrations of glucans were then estimated by subtracting the quantity of free monomeric glucose from the total glucose content.

## Results

### Knocking out the lactate and acetate pathways in Thermoanaerobacterium strain AK17

**Fig. 1:**
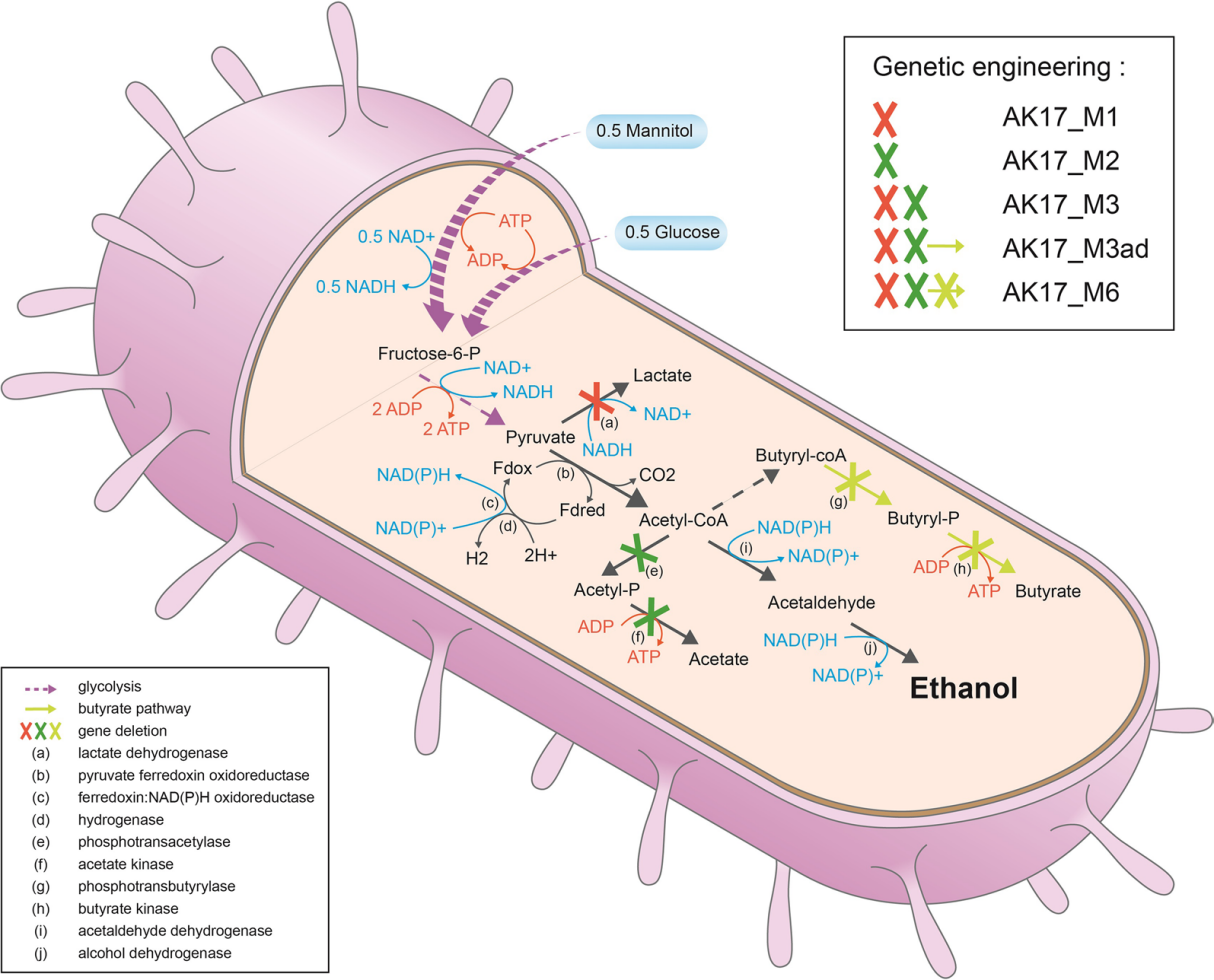
*Schematic picture overviewing metabolic pathways in Thermoanaerobacterium AK17.* The pathways for production of ethanol, acetate, lactate and butyrate in *Thermoanaerobacterium* AK17 are shown. Positions in the pathways where genes have been deleted are indicated by crosses (red cross: *ldh* knockout; green cross: *ack/pta* knockout; yellow cross: *bk/ptb* knockout) and the designations of the corresponding engineered strains are shown in the box in the upper right corner. The enzymes responsible for different steps in the pathways are indicated by letters (**a**–**j**) in the pathways, and the corresponding enzymes are shown in the explanatory box in the lower left corner of the figure

The first step in the genetic engineering of strain AK17 was to knockout the genes in the pathways leading to production of lactate and acetate during fermentation (Fig. 1), with the aim of obtaining a strain that would only produce ethanol. The lactic acid production pathway was knocked out by deleting the lactose dehydrogenase *ldh* gene, and the acetic acid production pathway was knocked out by deleting both the acetate kinase *ack-* and phosphotransacetylase *pta* genes.

Cloning and transformation procedures were performed as described earlier, and colonies growing on plates containing selective medium following transformation were verified for the appropriate deletion/insertion using PCR and sequencing. One colony was chosen for each mutation, resulting in isolation of the strains AK17_M1 (*Δldh:erm*) with the *ldh* gene deleted (Lac-), and AK17_M2 (*Δack/pta:kan*), with both the *ack* and *pta* genes deleted (Ace-). In a second step, the acetate production pathway in strain AK17_M1 was knocked out following the same procedure as for AK17_M2, leading to the third strain AK17_M3 (*Δldh:erm, Δack/pta:kan*) which represents a double pathway knockout (Lac-/Ace-) expected to remove production of both lactate and acetate.

**Fig. 2:**
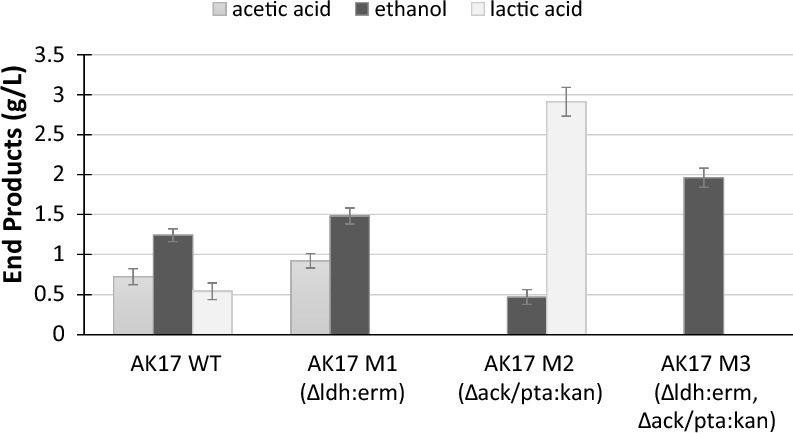
End product fromation of AK17 (wild type = WT), and the new engineered strains, in media containing 4.5 g/L glucose. Samples were taken at 0 h (inoculation) and after 24 h (end of fermentation). Acetic acid (dark gray), ethanol (black) and lactic acid (light gray). The absence of a bar indicates the product was below the detection limit. Data represent the average of three replicate experiments. Standard deviations are shown as error bars

The fermentation products of the wild-type strain (AK17) and the three new strains (AK17_M1, AK17_M2, and AK17_M3) were subsequently analysed from glucose-grown cultures to verify their corresponding phenotypes (Fig. 2, Additional file 3: Table S2). The *Δldh* (Lac-) mutant (strain AK17_M1) produced undetectable amounts of lactic acid, with a 20% increase of both the produced acetic acid and ethanol compared to the wild-type strain. The *Δack/pta (*Ace-) mutant (strain AK17_M2) showed undetectable amounts of acetic acid, with more than a fivefold increase in the produced lactic acid combined with an unwanted almost threefold decrease in the produced ethanol. Finally, the double pathway mutant (Lac-/Ace-) (strain AK17_M3) produced undetectable amounts of both lactic and acetic acid with a 58% increase of the produced ethanol compared to the wild-type strain, reaching a yield of 0.45 g_eth_/g_glucose_, which is around 88% of the maximum theoretical yield. The final cell densities (OD_600nm_ after 24 h) in the cultures were approximately 20% lower for the engineered strain AK17_M2 (*Δack/pta:kan*) and AK17_M3 (*Δldh:erm, Δack/pta:kan*) compared to wild-type AK17. No difference in cell density was, however, observed between the wild-type and strain AK17_M1 (*Δldh:erm*).

### Reversion of acetic acid production in Thermoanaerobacterium AK17_AM3 (Δldh:erm Δack/pta:kan)

After repeated subculturing (~ 10 times), strain AK17_M3 started to produce acetic acid, in the same amounts as the wild-type strain. Moreover, there was no longer any difference in final cell density between the wild-type and the reverted acetate producing strain. After isolation, this newly adapted strain was called AK17_M3ad. The genome of strain AK17 was analysed to understand the mechanism of this adaptation and resulted in the identification of two potential gene candidates that could compensate for the loss of the phosphotransacetylase (PTA) and acetate kinase (AcK) in AK17_M3, i.e. the genes encoding the phosphotransbutyrylase (PTB) (EC 2.3.1.19) and butyrate kinase (BK) (EC 2.7.2.7), respectively (Fig. 1). PTA (EC 2.3.1.8) and PTB belong to the same family of acyltransferases, and this is also the case for AcK (EC 2.7.2.1) and BK, which belong to the same family of phosphotransferases. Also, it has previously been reported that butanoate pathway enzymes are able to compensate for lost acetate production (Kuit et al. [39]). Subsequently, the hypothesis was formed that both PTB and BK have a dual specificity, being able to act in the acetate as well as the butyrate production pathway. Butyrate was not detected as a product of fermentation in AK17, but the hypothesis is that the activities of PTB and BK are induced in AK17_M3ad. To verify this hypothesis, several enzymatic activities were compared from the crude extracts of AK17, AK17_M3, and AK17_M3ad, as described in the Material and methods section.

**Fig. 3:**
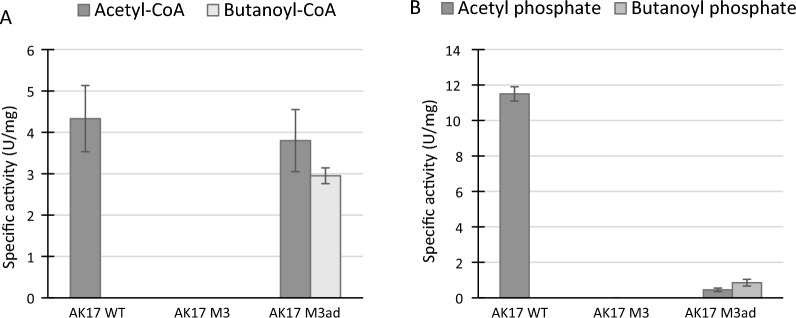
Activity assay of PTA and PTB (**A**) and AcK and BK (**B**) from strain AK17 (wild-type = WT) and the two other mutants AK17_M3 and AK17_M3ad. **A** Acetyl-CoA (dark gray) and Butanoyl-CoA (light gray). **B** Aceyl phosphate (dark gray) and Butanoyl phosphate (light gray). The absence of a bar indicates the product was below the detection limit Data represent the average of three replicate experiments. Standard deviations are shown as error bars

First, acyltransferase activities were tested using acetyl-CoA or butanoyl-CoA as substrates (Fig. 3A). The AK17 crude extract showed activity on acetyl-CoA, and almost no activity on butanoyl-CoA. The original AK17_M3 crude extract did not show any significant activity on either substrate, but on the contrary, the AK17_M3ad crude extract showed activity on both substrates. The activity on acetyl-CoA was even comparable to that of the wild-type strain.

The phosphotransferase activities were tested using the substrates acetyl phosphate and butanoyl phosphate (Fig. 3B). The AK17 crude extract had high activity on acetyl phosphate and no significant activity on butanoyl phosphate (100-fold less). The original AK17_M3 crude extract did not show any significant activity on either substrate, while the AK17_M3ad crude extract showed activity on both butanoyl phosphate and acetyl phosphate, but the activity was rather low compared to the activity of the wild-type strain (20-fold difference).

Both assays showed that there is increased activity of PTB and BK in strain AK17_M3ad and that both enzymes are also active on acetate precursors. These results support the hypothesis that PTB and BK were produced to alleviate the absence of PTA and AcK, restoring the acetic acid production in strain AK17_M3ad.

### Knockout of the butyrate pathway in strain AK17_M3 (*Δldh:erm Δack/pta:kan)*

**Fig. 4:**
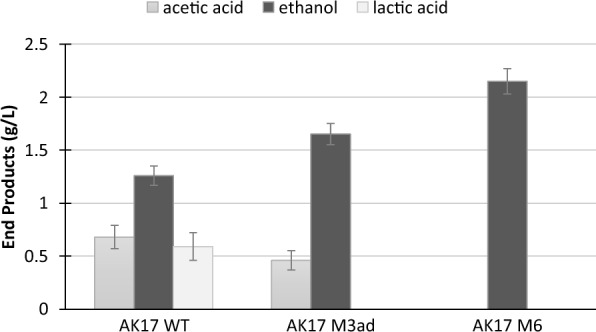
End product formation of AK17_WT and strains AK17_M3ad and AK17_M6, on media containing 4.5 g/L glucose. Samples were taken at 0 h (inoculation) and after 24 h (end of fermentation). Acetic acid (dark gray), ethanol (black) and lactic acid (light gray). The absence of a bar indicates the product was below the detection limit. Data represent the average of three replicate experiments. Standard deviations are shown as error bars

The next step in the engineering of strain AK17 was to eliminate the two relevant butyrate pathway genes, encoding PTB and BK, respectively. This was made using the pyrF recycling marker system strategy (Additional file 2: Figure S1, as described in the Material and methods section), to be able to re-use the erythromycin selection. Subsequently, the triple pathway mutant strain AK17_M6 (*Δldh Δack/pta:kan Δbk/ptb:erm*) was obtained and subcultured more than ten times to confirm its stability. The final cell density of AK17_M6 was in the same range as observed for AK17_M3 (*Δldh:erm, Δack/pta:kan*) prior to the reversion of acetate production. Analysis of the fermentation products of the wild-type strain AK17, and the two mutant strains AK17_M3ad and AK17_M6 in glucose-grown cultures was performed in parallel to verify their corresponding phenotypes (Fig. 4, Additional file 4: Table S3).

Strain AK17_M3ad produced acetic acid in almost similar amounts as the wild-type, but despite an increased ethanol production compared with the wild-type, it produced lower amounts of ethanol as compared with the original AK17_M3 strain. Strain AK17_M6 with the three deleted pathways, however, produced ethanol, as the sole volatile end product metabolite, at a high yield of 0.47 g_ethanol_/g_glucose_, close to the theoretical yield (91%).

### Fermentation on other substrates

The fermentation capabilities of the wild-type strain and the ethanologenic strain AK17_M6 were then investigated on substrates present in 3^rd^ generation (seaweed) biomass, i.e. mannitol or a mixture of glucose and mannitol. Both strains were grown in anaerobic flasks containing media supplemented with either 4.5 g/L glucose or mannitol, or with 4.5 g/L glucose and mannitol (Table 2). On a single substrate, the wild-type strain AK17 produced more ethanol from mannitol than from glucose (35% increase in yield), but was unable to utilize all the supplemented mannitol (65% consumption) while all glucose was consumed. There was no difference in the ethanol yield from growth on the two substrates (mannitol or glucose) for strain AK17_M6 (90% of the theoretical yield in both cases), but again the strain was not capable of utilizing all the mannitol. By mixing both substrates, the overall ethanol yield was higher, as compared to the yield from glucose alone (62% of the theoretical yield for the wild-type and 92% of the theoretical yield for strain AK17_M6). Both strains consumed all the glucose, but only a part of the mannitol.

**Table 2:** Fermentation profiles of AK17_WT and AK17_M6, in medium containing 4.5 g/L glucose or 4.5 g/L mannitol or both 4.5 g/L glucose and mannitol. Samples were taken at 0 h (inoculation) and after 24 h (end of fermentation).

				Fermentation products (g/L)			
Strains and conditions		Glucose consumed (g/L)	Mannitol consumed (g/L)	Ethanol	Acetic acid	Lactic acid	Ethanol yield (g_eth_/g_substrate_)
AK17 WT	Glucose	4.61 ± 0.16	-	1.31 ± 0.15	0.82 ± 0.09	0.65 ± 0.08	0.28 (55%)
	Mannitol	–	2.93 ± 0.12	1.10 ± 0.14	0.19 ± 0.05	0.12 ± 0.04	0.38 (73%)
	Glucose + mannitol	4.51 ± 0.15	2.37 ± 0.13	2.19 ± 0.12	1.10 ± 0.11	0.82 ± 0.07	0.32 (62%)
AK17 M6	Glucose	4.64 ± 0.13	-	1.94 ± 0.14	ND	ND	0.46 (91%)
	Mannitol	–	2.78 ± 0.18	1.27 ± 0.11	ND	ND	0.47 (92%)
	Glucose + mannitol	4.48 ± 0.18	2.25 ± 0.09	3.16 ± 0.16	ND	ND	0.47 (92%)

Data represent the average of three replicate experiments

Finally, the fermentative capability of strains AK17_WT and AK17_M6 was tested on a seaweed hydrolysate from *Laminaria digitata* [29] under two conditions (using 50% and 100% hydrolysate, respectively). The carbohydrate content of the *L. digitata* hydrolysate was: 10 g/L glucose, 5 g/L mannitol, 10 g/L glucans, and 1.2 g/L lactic acid, where lactic acid was judged as a contamination introduced during enzymatic processing, as described by Hou and co-workers [31].

**Fig. 5:**
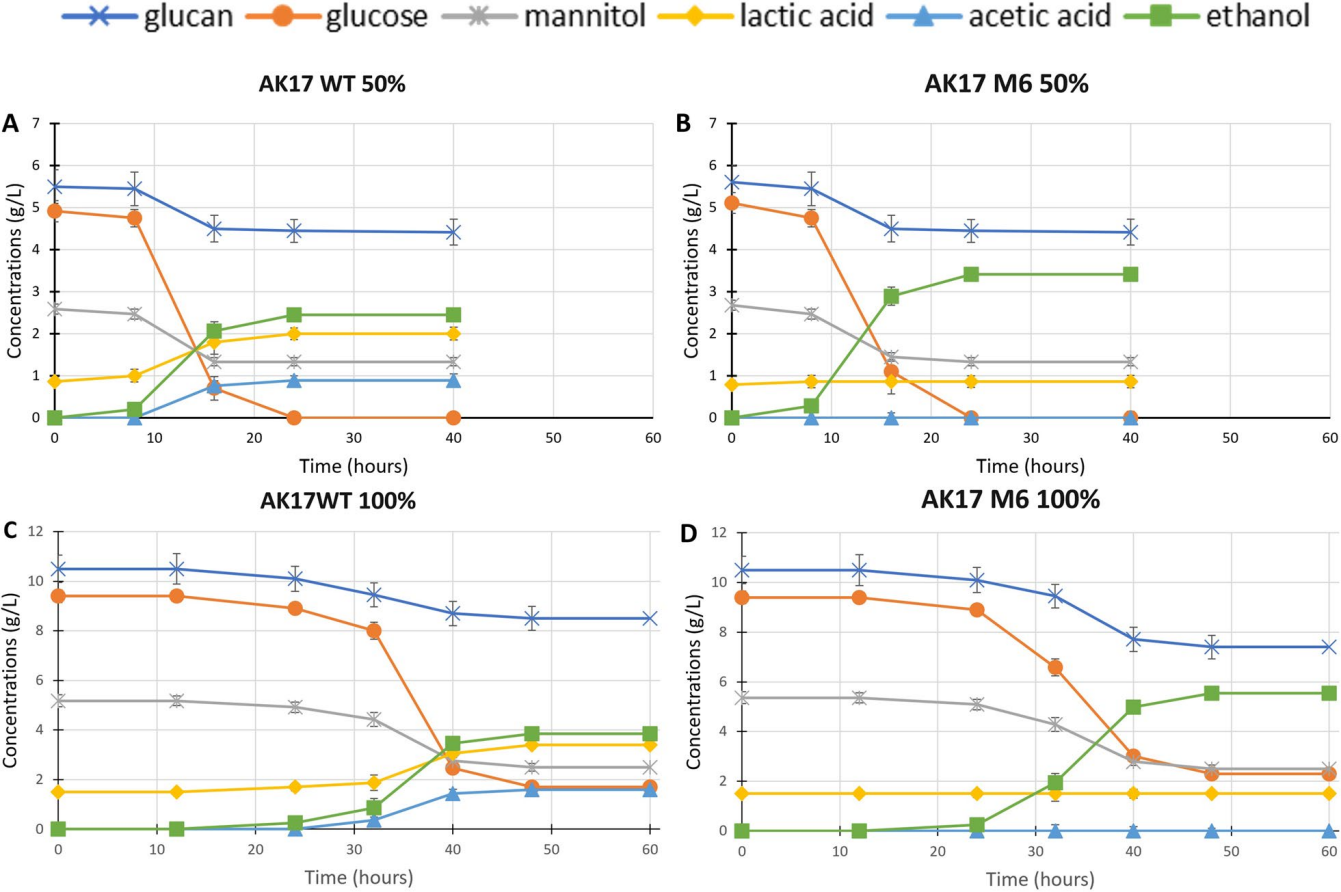
Fermentation kinetics of AK17 (WT) and AK17_M6, in 50% or 100% L. digitata hydrolysate. Fermentations were carried out for 40 h with 50% hydrolysate, for both AK17 (**A**) and AK17_M6 (**B**) and samples were taken at different times between 0 h (inoculation) and 40 h (end of fermentation). Fermentations were carried out for 60 h with 100% hydrolysate, for both AK17 (**C**) and AK17_M6 (**D**) and samples were taken at different times between 0 h (inoculation) and 60 h (end of fermentation). Glucan (blue cross), glucose (orange circle), mannitol (grey cross), lactic acid (yellow diamond), acetic acid (blue triangle) and ethanol (green square). Data represent the average of three replicate experiments. Standard deviations are shown as error bars

Both strains were able to grow under both conditions (Table 3). In cultivations using diluted hydrolysate (50%) all the monomeric glucose, 20% of the glucan and 50% of the mannitol were consumed, resulting in conversion of 56% of the total carbon sources (50% hydrolysate, Table 3). The duration of the fermentation on the 50% hydrolysate was 24 h (from inoculation), with a growth phase lasting around 16 h (Fig. 5A and B). It is also interesting to note that strain AK17_M6 showed a growth phase that corresponded to the growth phase of the wild-type strain. On the undiluted hydrolysate (100% hydrolysate, Table 3) strains AK17_WT and AK17_M6 used 82% and 75% of the glucose, 20% and 29% of the glucan, 47% and 43% of the mannitol, respectively. A slightly reduced amount of the carbohydrates was converted compared with the diluted hydrolysate (51% of the carbon sources in the hydrolysate), mainly due to the lower conversion of the available glucose (Fig. 5C and D). The fermentations on 100% hydrolysate took longer time than the corresponding cultivations on diluted hydrolysate (mainly due to a lag phase of 12 h), finishing 48 h after inoculation, with a growth phase lasting around 24 h (Fig. 5C and D).

**Table 3:** Fermentation profiles of AK17_WT and AK17_M6, on either 50% or 100% L. digitata hydrolysate

									Fermentation products (g/L)				
Strains and conditions			Glucose (g/L)	Mannitol (g/L)		Glucan (g/L)		Ethanol		Acetic acid	Lactic acid		Ethanol yield (g_eth_/g_substrate_)
AK17 WT	50% hydrolysate	Initial	5.1 ± 0.15	2.8 ± 0.08		5.6 ± 0.21							
		After 40 h	ND	1.4 ± 0.06		4.5 ± 0.2		2.21 ± 0.12		1.30 ± 0.11	2.26 ± 0.07		0.29 (57%)
	100% hydrolysate	Initial	9.5 ± 0.21	4.9 ± 0.07		10.6 ± 0.35							
		After 60 h	1.7 ± 0.13	2.6 ± 0.07		8.5 ± 0.31		3.82 ± 0.14		1.54 ± 0.09	3.08 ± 0.11		0.31 (61%)
AK17 M6	50% hydrolysate	Initial	5.2 ± 0.12	2.7 ± 0.05		5.5 ± 0.23							
		After 40 h	ND	1.45 ± 0.08		4.6 ± 0.19		3.41 ± 0.16		ND	ND		0.46 (91%)
	100% hydrolysate	Initial	9.3 ± 0.25	4.9 ± 0.09		10.4 ± 0.38							
		After 60 h	2.3 ± 0.14	2.8 ± 0.06		7.4 ± 0.32		5.54 ± 0.12		ND	ND		0.45 (89%)

Data represent the average of three replicate experiments

Regarding the fermentation products, strain AK17_WT produced ethanol as the main fermentation product, but in addition to this, lactic acid and acetic acid were also produced (Table 3). The ethanol yield under both conditions (~ 60%) is close to what was observed in the cultivation on glucose and mannitol (Table 2). In the cultivation on undiluted hydrolysate, the proportion of lactic acid was increased, as compared to the product profile obtained during growth on the diluted hydrolysate, whereas the production of acetic acid was similar (100% hydrolysate, Table 3).

For strain AK17_M6, only ethanol was produced, in yields corresponding to those observed in the experiments using glucose and mannitol as substrates (Table 2), reaching around 90% of the maximum theoretical yield, and with an ethanol productivity of 0.32 g/L/h (Fig. 5B and D).

## Discussion

### Knockout of the lactate and acetate pathways in strain AK17

Initially the genetic engineering of AK17, to improve ethanol production, entailed individually knocking out enzymes in the lactic acid and acetic acid pathways, to eliminate byproducts of the fermentation. When only the *ldh* gene was deleted, strain AK17_M1 produced 20% more ethanol than the wild-type strain and did not produce lactate. This is consistent with previous studies, where similar deletions led to a 10% increase in ethanol production in *Thermoanaerobacterium saccharolyticum* [12], and a 23% increase in *Clostridium thermocellum* [13]. The ethanol:acetate ratio was also similar to that of the wild-type after deletion of the *ldh* gene. However, when the acetate production pathway was knocked out, by deleting the *ack/pta* genes, strain AK17_M2 showed a 60% decrease in ethanol and a fivefold increase in lactic acid production. Similarly, in C. *thermocellum*, deletion of the *pta* gene increased the production of lactic acid by 60%, whereas in *T. saccharolyticum* the same deletion did not affect the lactic acid production [12, 13].

This metabolic shift reflects the trade-off between redox potential balance and energy considerations during fermentation, which affects the use of alternative metabolic pathways, at positions in metabolism where alternative routes are possible, such as the route from pyruvate, or from acetyl-CoA (Fig. 1). Through the lactate pathway, electrons are added to pyruvate from reduced NADH generated during glycolysis, allowing the overall redox potential of the glycolysis to be balanced. But on the other hand, the metabolic pathways leading to acetic acid and ethanol production involve the further oxidation of pyruvate to acetyl-CoA and reduced ferredoxin (Fig. 1, reactions b-c-d). The acetyl-CoA is then either converted to acetate, producing an additional ATP but no redox equivalents, or converted to ethanol with the regeneration of two cofactors. The additional reduction of electron carriers versus the production of extra energy in these pathways creates metabolic key points which control the yields of the fermentation products. In strain AK17_M2, the deletion of the ack/pta genes eliminates the energy advantage associated with acetyl-CoA conversion to acetate. The hypothesis would be that pyruvate is predominantly fermented to lactate, as the redox potential is easily balanced using this pathway. And as a consequence, the acetyl-CoA formation decreases, and so does the ethanol. Deeper investigations in the pyruvate metabolism and cofactor usage [37] would, however, be needed to confirm this hypothesis.

When both the acetate and lactate pathways were knocked out, strain AK17_M3 produced only ethanol, with a 55% increase compared to the wild type and without detectable organic acids (neither lactic acid, acetic acid nor other potential metabolites). This is higher than the 40% increase obtained in *T. saccharolyticum*, but similar to the 56% increase in ethanol production obtained in *C. thermocellum*. The ethanol yield for strain AK17_M3 reached 0.45 g ethanol per g glucose, similar to the yield obtained by the engineered strain *T. saccharolyticum* M0355 on cellobiose [24]. Strain AK17_M3 was, however, not stable, as discussed further below. These results highlight the delicate balance between redox potential equilibrium and energy production during the fermentation. Specifically, the choice between lactate and acetyl-CoA formation from pyruvate plays a pivotal role in maintaining this balance. Consequently, mutations can lead to imbalances in carbon and electron fluxes, which are specific to the organism and need to be addressed accordingly [13, 16, 38].

### Acetate revertant and butyrate pathway knockout in strain AK17_M3

After repeated subculturing, strain AK17_M3 started to produce acetic acid again, in the same amounts as the wild-type strain, although deletions of both *ack* and *pta* genes were still present. An ATP molecule is formed for each acetyl-CoA going through the acetic acid pathway. By knocking out this pathway, the cells produced less energy and therefore reached a lower optical density as compared to the wild-type strain (data not shown). The lower cell mass obtained indicated that these cells were under selective pressure to adapt to new ways of producing acetate. By restoring acetic acid production, and therefore gaining additional ATP, the cells may have gained a growth advantage over strain AK17_M3. It is not uncommon that genetically modified strains evolve spontaneously, especially when cultivated repeatedly, leading to various unwanted phenotypes [39, 40]. These ancillary mutations emphasize the need for better control during metabolic engineering, especially when working with versatile organisms such as extremophiles. In this study, the versatility of strain AK17 was illustrated by the spontaneous evolution of the strain AK17_M3ad, leading to upregulation of the BK and PTB enzymes, belonging to the same family as the AcK and PTA enzymes, respectively, and allowing a regained production of acetic acid.

Finally, it was logical to knock out the butyrate pathway in strain AK17_M3 to avoid any possible reversion. At that point, as mentioned earlier, a new strategy was needed for further engineering, as no additional thermostable antibiotic was available for selection. By using the *pyrF* recycling marker system, which has been studied extensively, it was possible to rapidly reuse erythromycin selection, and later continue with this system for further engineering [18, 24, 41]. For the engineering aspect, it is important to note that CRISPR genome editing systems have not yet been fully developed for genetic manipulation of species within the genus *Thermoanaerobacterium* and are only beginning to emerge [42]. Recent advances are, however, encouraging and could lead to make the genetic and metabolic engineering of thermophilic organisms easier [23, 43]. The newly constructed strain AK17_M6 (*Δldh Δack/pta:kan Δbk/ptb:erm*) produced ethanol as the only product, with a yield of 90% compared to the theoretical maximum.

### Fermentation on mixed sugars and seaweed hydrolysate

Fermentation capabilities of the wild-type strain AK17 and the engineered strain AK17_M6 were investigated on mannitol or on a mixture of glucose and mannitol, as both components are significant in seaweeds, which are third-generation biomasses. While strain AK17 is capable of consuming all the glucose, utilization of mannitol was always limited, with a consumption of 50 to 65% depending on the culture conditions. Mannitol has a lower redox potential (− 0.32 V) than glucose (− 0.17 V), meaning that it needs an extra oxidation step before entering the glycolysis, which is accompanied by the formation of extra NADH. This extra reducing power has been shown to direct carbon flux towards ethanol production [44]. So, even though mannitol should have changed the redox balance, allowing more ethanol to be produced, it seems that there is an imbalance that prevents AK17 from utilizing all of it. In the wild-type strain, use of mannitol increased the ethanol yield (up to 20%). Similar results were observed in *Thermoanaerobacter mathranii* with a consumption of 80% of the available mannitol, leading to a 22.6% increase in ethanol yield compared with fermentation on glucose [45]. The ethanol yield on mannitol reached 0.47 g/g mannitol, which is slightly higher than the 0.44 g/g yield obtained with *Defluviitalea phaphyphila* Alg1 on the same substrate [46], and higher than the 0.41 g/g yield obtained with the engineered strain *E. coli* KO11 [10]. In mixed sugar fermentations, the new strain AK17_M6 was able to use all added glucose and part of the mannitol to produce ethanol at high yield (90%), which makes it a good candidate for the fermentation of seaweed hydrolysate.

*Thermoanaerobacterium* AK17 was previously shown to be able to grow on a *L. digitata* hydrolysate, where it consumed glucose, mannitol, and part of the glucan fraction, while producing ethanol and organic acids [29]. In this study, the ethanologenic strain AK17_M6 was used to ferment the same hydrolysate and its performance was compared to the performance of the wild-type strain. Although having a similar substrate consumption profile, strain AK17_M6 produced ethanol as the only detectable product at high yield (89% of the maximum theoretical yield). Also, with a growth phase of 24 h, the ethanol productivity of AK17_M6 reached 0.32 g/L/h. These results are promising when comparing to other organisms and studies. For example, *E. coli* KO11 was engineered for mannitol utilization, and was able to ferment *S. japonica* (after acid pre-treatment and enzymatic hydrolysis) hydrolysates reaching an ethanol yield of 0.4 g/g of sugars, with an ethanol productivity of 0.22 g/L/h [10].

For yeast, bioethanol yields of 71–76% are reported using *Sargassum* spp as biomass [47, 48], and *S. cerevisiae* KCTC 7906 achieved bioethanol yields of 74–84% using the red seaweed *Gelidium amansii* [49]. *S. cerevisiae* AM1 was also engineered to use both alginate and mannitol, but with a lower yield of 32% [11]. More recently, the marine yeast *Wickerhamomyces anomalus* M15 was shown to produce ethanol at high yields from synthetic seaweed medium and was tested for its high tolerance toward stress (salt, substrate loading), but was unable to use mannitol [50, 51]. The only other thermophilic organism known to ferment seaweed is *Defluviitalea phaphyphila* Alg1, which was shown to produce ethanol from brown macroalgae (kelp powder) with a similar yield as AK17_M6, but at a lower productivity of 0.13 g/L/h [46].

Additionally, several other sources of non-food biomass, including wheat straw, sugarcane bagasse, and saw dust [8, 52] have also been studied for use in the production of fermentative biofuels, in addition to seaweed. These biomasses are composed of a mixture of carbohydrates, but mainly of structural polymers (cellulose, hemicellulose, pectin, and lignin) that comprise the cell wall [53]. The sugar residues of hemicellulose also contain a varying mixture of hexoses (e.g., glucose, mannose, galactose) and pentoses (e.g., arabinose and xylose). Strain AK17 was previously shown to utilize all of these residues, and also pectin polymers [25, 27], as well as oligosaccharides of β-1,3 glucans as a carbohydrate source [29]. These results highlight that the newly engineered strain AK17_M6 could be used to produce ethanol from both second and third generation biomass, making it an efficient and versatile fermentative microorganism producing only ethanol from a variety of substrates.

## Conclusion

In this study, the thermoanaerobe *Thermoanaerobacterium* AK17 was selected for engineering, being a versatile fermentative bacterium able to utilize second and third generation biomass as substrates, producing ethanol, acetate and lactate. The microorganism was successfully engineered to an ethanologenic strain termed *Thermoanaerobacterium* AK17_M6, producing ethanol close to the theoretical yield (90%), as the sole volatile metabolite, by knocking out the lactate, acetate as well as the butyrate production pathways. The latter pathway was targeted to obtain a stable strain, as repeated cultivation led to reversion to acetate production, via upregulation of related enzymes from the butyrate pathway. The developed strain AK17_M6 successfully fermented brown seaweed (*L. digitata*) hydrolysate to ethanol reaching a yield of 0.45 g/g substrate, using mannitol, laminarin-derived glucose and short laminari-oligosaccharides from the hydrolysate as substrates. This makes the engineered strain promising for biorefinery applications. Further engineering work would be helped by access to novel CRISP-R tools for thermophiles, for example targeting tolerance to high substrate and product loadings.

### Supplementary Information

**Additional file 1: Table S1.** Primers used for the construction of all cassettes. Bold bases denote an overlap with a region to which the amplified sequence was spliced to using SOE-PCR.

**Additional file 2: Figure S1.** Scheme of recycling of the pyrF marker.

**Additional file 3: Table S2.** Fermentation profiles of the wild-type AK17 (WT) and the new engineered strains, in media containing 4.5 g/L glucose. Data represent average of three replicate experiments.

**Additional file 4: Table S3.** Fermentation profiles of wild type (WT) AK17, the new adapted strain AK17_M3ad and the engineered strain AK17_M6, on media containing 4.5 g/L glucose. Data represent average of three replicate experiments.

## Data Availability

Data will be made available on request.
