# Supplementary material for: Metabolic engineering of Thermoanaerobacterium AK17 for increased ethanol production in seaweed hydrolysate
Source: Biotechnol Biofuels Bioprod. 2023 Sep 11;16:135. doi: 10.1186/s13068-023-02388-y (PMC10496261; doi:10.1186/s13068-023-02388-y)
Supplement: Supplementary file 1 — Additional file 1: Table S1. Primers used for the construction of all cassettes. Bold bases denote an overlap with a region to which the amplified sequence was spliced to using SOE-PCR. [file 13068_2023_2388_MOESM1_ESM.pdf]

Table S1. *Primers used for the construction of all cassettes. Bold bases denote an overlap with a region to which the amplified sequence was spliced to using SOE-PCR.*

**Primer**	**Description**	**Sequence (5´-3´)**
5’ldh-upstream-erm-f	Construction of the insertion cassette of the erm gene into the ldh locus, to be ligated into pUC plasmid	**CCATGATTACGAATTCGAGCTCGGTACCC**GAGAGTGTCGTAGCTCATGTAGTC
5’ldh-upstream-erm-r		**CTTTATGTTCTTATTCATTAC**ATCGCCTCCTAAATAATATATATTTTC
3’ldh-downstream-erm-f		**GTTATTCAATGGAAGGAAGTAA**GGATCCAAGTGTCATGGC
3’ldh-downstream-erm-r		**CTGCAGGTCGACTCTAGAGGATCCC**CGGATTAGTTATGTTTATG
erm_ldh-f		**TTATTTAGGAGGCGATGTA**ATGAATAAGAACATAAAGTACAGTCAG
erm_ldh-r		**ATCTGCCATGACACTTGGATCC**TTACTTCCTTCCATTGAATAAC
5’ak/pta-upstream-f	Construction of the insertion cassette of the kan gene into the ak/pta locus, to be ligated into pUC plasmid	**CCATGATTACGAATTCGAGCTCGGTACCC**GCTGCTGAGATAGTCC
5’ak/pta-upstream-r		**AACATCCTCCTCTACAATT**AAGTTATTATGCATTAGGCG
3’ak/pta-downstream-f		**GCATACCATTTTGAGAATT**TTGACACAAGCATGGG
3’ak/pta-downstream-r		**CTGCAGGTCGACTCTAGAGGATCCC**TACTCTTTACAATCTTTTC
Kan-f		**CGCCTAATGCATAATAACTT**AATTGTAGAGGAGGATGTT
Kan-r		**CCCATGCTTGTGTCAA**AATTCTCAAAATGGTATGC
5’pyrF-upstream-f	Construction of the deletion cassette of the pyrF gene into the pyrF locus, to be ligated into pUC plasmid	**CCATGATTACGAATTCGAGCTCGGTACCC**ACGCCGCACAATCTA
5’pyrF-upstream-r		**AACGTATTCCATATTTCACC**ATTAACACCTCTTATAAATATTTATTC
3’pyrF-downstream-f		**TATTTATAAGAGGTGTTAAT**GGTGAAATATGGAATACGTT
3’pyrF-downstream-r		**CTGCAGGTCGACTCTAGAGGATCCC**CATTTGCATTGGACCACATC
5’ldh-upstream-pyrF-f	Construction of the insertion cassette of the pyrF gene into the ldh locus, to be ligated into pUC plasmid	**CCATGATTACGAATTCGAGCTCGGTACCC**GAGAGTGTCGTAGCTCATGTAGTC
5’ldh-upstream-pyrF-r		**GCTATTGTGGCCGTCATTTG**AAAATATATATTATTTAGGAGGCGATGTA
3’ldh-downstream-pyrF-f		**CAAGAATATGGAAGGTGCTTTCGAT**AATTGTCAAATATTTTTGAG
3’ldh-downstream-pyrF-r		**CTGCAGGTCGACTCTAGAGGATCCC**CCGGATTAGTTATGTTTAT
pyrF-ldh-f		**TACATCGCCTCCTAAATAATATATATTTT**CAAATGACGGCCACAATAGC
pyrF-ldh-r		**CTCAAAAATATTTGACAATT**ATCGAAAGCACCTTCCATATTCTTG
5’ldh-upstream-f	Construction of the deletion cassette of the pyrF gene into the ldh locus, to be ligated into pUC plasmid	**CCATGATTACGAATTCGAGCTCGGTACCC**GAGAGTGTCGTAGCTCATGTAGTC
5’ldh-upstream-r		**CTCAAAAATATTTGACAATTG**AAAATATATATTATTTAGGAGGCGATGTA
3’ldh-downstream-f		**TACATCGCCTCCTAAATAATATATATTTTC**AATTGTCAAATATTTTTGAG
3’ldh-downstream-r		**CTGCAGGTCGACTCTAGAGGATCCC**CCGGATTAGTTATGTTTAT
5’ptb/bk-upstream-f	Construction of the insertion cassette of the pyrF gene into the ptb/bk locus, to be ligated into pUC plasmid	**CAAGAATATGGAAGGTGCTTTCGATG**TTTTCGGATAATTTGATACATGC
5’ ptb/bk-upstream-r		**CACTCCTCTTTTTAATATTTTAGG**CAATTTCAACACGCAACATATCTTC
3’ ptb/bk-downstream-f		**GAAGATATGTTGCGTGTTGAAATTG**CCTAAAATATTAAAAAGAGGAGTG
3’ ptb/bk-downstream-r		**CTGCAGGTCGACTCTAGAGGATCCC**CCAACATTCCCAACACCTTGAATAGC
pyrF-btk/bk-f		**CCATGATTACGAATTCGAGCTCGGTACCC**CAAATGACGGCCACAATAGC
pyrF-btk/bk-r		**GCATGTATCAAATTATCCGAAAAC**ATCGAAAGCACCTTCCATATTCTTG
M13-f	universal primer	GTTTTCCCAGTCACGAC
M13-r	universal primer	AACAGCTATGACCATG
