# Supplementary figures and images for: Metabolic engineering of Thermoanaerobacterium AK17 for increased ethanol production in seaweed hydrolysate
Source: Biotechnol Biofuels Bioprod. 2023 Sep 11;16:135. doi: 10.1186/s13068-023-02388-y (PMC10496261; doi:10.1186/s13068-023-02388-y)

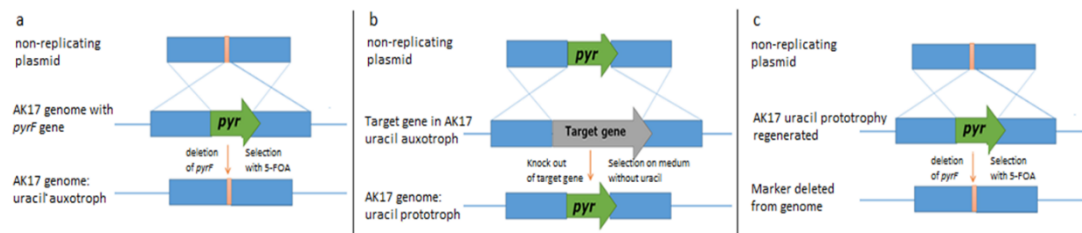

**Figure S1.** Scheme of recycling of the *pyrF* marker.

Supplement: Supplementary file 2 — Additional file 2: Figure S1. Scheme of recycling of the pyrF marker. [file 13068_2023_2388_MOESM2_ESM.pdf]
