# Supplementary material for: Metabolic engineering of Thermoanaerobacterium AK17 for increased ethanol production in seaweed hydrolysate
Source: Biotechnol Biofuels Bioprod. 2023 Sep 11;16:135. doi: 10.1186/s13068-023-02388-y (PMC10496261; doi:10.1186/s13068-023-02388-y)
Supplement: Supplementary file 3 — Additional file 3: Table S2. Fermentation profiles of the wild-type AK17 (WT) and the new engineered strains, in media containing 4.5 g/L glucose. Data represent average of three replicate experiments. [file 13068_2023_2388_MOESM3_ESM.pdf]

Table S2. *Fermentation profiles of the wild type AK17 (WT) and the new engineered strains, in media containing 4.5g/L glucose. Data represent average of three replicate experiments.*

Strains	Glucose consumed (g/L)	Fermentation products (g/L)			Ethanol yield ($g_{ethanol}/g_{glucose}$)
		Ethanol	Acetic acid	Lactic acid	
AK17 WT	4.55 ± 0.32	1.24 ± 0.08	0.72 ± 0.10	0.54 ± 0.10	0.27 (53%)
AK17 M1 (*Δldh:erm*)	4.48 ± 0.21	1.48 ± 0.10	0.92 ± 0.09	ND	0.33 (65%)
AK17 M2 (*Δack/pta:kan*)	4.51 ± 0.23	0.47 ± 0.09	ND	2.91 ± 0.18	0.10 (20%)
AK17 M3 (*Δldh:erm Δack/pta:kan*)	4.38 ± 0.27	1.96 ± 0.12	ND	ND	0.45 (88%)
