# Supplementary material for: Metabolic engineering of Thermoanaerobacterium AK17 for increased ethanol production in seaweed hydrolysate
Source: Biotechnol Biofuels Bioprod. 2023 Sep 11;16:135. doi: 10.1186/s13068-023-02388-y (PMC10496261; doi:10.1186/s13068-023-02388-y)
Supplement: Supplementary file 4 — Additional file 4: Table S3. Fermentation profiles of wild type (WT) AK17, the new adapted strain AK17_M3ad and the engineered strain AK17_M6, on media containing 4.5 g/L glucose. Data represent average of three replicate experiments. [file 13068_2023_2388_MOESM4_ESM.pdf]

Table S3. *Fermentation profiles of wild type (WT) AK17, the new adapted strain AK17_M3ad and the engineered strain AK17_M6, on media containing 4.5g/L glucose. Data represent average of three replicate experiments.*

Strains	Glucose consumed (g/L)	Fermentation products (g/L)			Ethanol yield ($g_{ethl}/g_{glucose}$)
		Ethanol	Acetic acid	Lactic acid	
AK17 (WT)	4.42 ± 0.14	1.26 ± 0.06	0.68 ± 0.04	0.59 ± 0.05	0.29 (56%)
AK17 M3ad (*Δldh:erm Δack/pta:kan*)	4.38 ± 0.27	1.65 ± 0.07	0.46 ± 0.09	ND	0.38 (74%)
AK17 M6 (*Δldh Δack/pta:kan Δbck/ptb:erm*)	4.62 ± 0.11	2.15 ± 0.12	ND	ND	0.47 (91%)
